# Current Usage of Traditional Chinese Medicine for Breast Cancer—A Narrative Approach to the Experiences of Women with Breast Cancer in Australia—A Pilot Study

**DOI:** 10.3390/medicines4020020

**Published:** 2017-04-21

**Authors:** Dianna Porter, Suzanne Cochrane, Xiaoshu Zhu

**Affiliations:** 1 National Institute of Complementary Medicine, Western Sydney University, Penrith 2751, Australia; S.Cochrane@westernsydney.edu.au; 2 School of Science and Health, Western Sydney University, Penrith 2751, Australia; X.Zhu@westernsydney.edu.au

**Keywords:** breast cancer, traditional Chinese medicine, acupuncture, patient experiences, Chinese herbal medicine, integrative care in oncology

## Abstract

**Background:** The use of Traditional Chinese Medicine (TCM) by breast cancer patients is growing. Few studies have examined the complexity of breast cancer survivors’ attitudes, lived experiences, barriers, and perceptions in using TCM as part of their treatment journey. This article examines breast cancer survivors’ experiences, perceptions of, and benefits (or not) in using TCM. **Methods:** Qualitative research, using semi-structured interviews, was the chosen methodology. **Results:** Participants used TCM as a form of self-help and as a complement, not an alternative, to standard care. Overall, 100% of the participants used acupuncture, 62% used Chinese herbal medicine, 23% used Qigong, and 23% used Chinese dietary therapy. Participants reported perceived outcomes and health benefits from TCM usage ranging from increased coping mechanisms, relieving stress and side-effects of standard treatment, the desire to be pro-active in the treatment journey, and to have a locus of control. Some cited the need to have “time-out” and the therapeutic relationship with the practitioner as being important. **Conclusion:** There is a clear need to understand breast cancer survivors’ needs for physical and psychological support as they aim to regain control over their life through their experience of illness. More studies are needed to measure and evaluate these outcomes and to help identify breast cancer survivors’ healthcare seeking behaviours, during and after the acute treatment stage that addresses their physical, emotional, and spiritual needs. These results aim to inform future research design and evaluate and develop support services that are patient-centred and focus on whole health outcomes, shared decision-making, and quality of life.

## 1. Introduction

Malignant breast cancer is a major health issue for women worldwide and has increased exponentially in the last three decades, particularly in the Western world. For Australian women, there is now a one in eight lifetime risk of being diagnosed with this type of malignancy [1]. With 15,000 Australian women diagnosed with breast cancer in 2009, this equates to a 300 per cent increase in the last three decades [2].

Improved earlier detection combined with improved treatment methods has resulted in decreased mortality in the last two decades [3]. Simultaneously, the use of Complementary and Alternative Medicine (CAM), in particular Traditional Chinese Medicine (TCM), has increased in western countries in this time frame, for healthy subjects and after a cancer diagnosis, as an adjunct to standard care [4,5,6,7,8]. Studies have found that 65% of cancer patients in Australia use some form of Complementary and Alternative Medicine (CAM), with TCM usage being as high as 36% and breast cancer survivors being amongst the highest users [4,5,6,7,9,10,11,12,13].

The theory of Traditional Chinese medicine is predicated on classical knowledge of several thousand years that has evolved over time and with clinical experience [14]. It is a complex holistic health system that is rooted in the ancient philosophy of Daoism, which emphasizes “naturalness” and man’s position between ‘heaven’ and ‘earth’ [15]. According to ancient theory “Qi” which is our life force energy, is carried throughout the body via meridians, which are the conduits of the body energy [15] “Qi” is responsible for physiological function: any abnormalities in the smooth flow of “Qi” causes a disharmony in the body which may result in a disruption to homeostasis and disease [16]. TCM aims to address these imbalances to enable the body to heal itself.

TCM is a complex set of interventions encompassing acupuncture, Chinese herbal medicine, moxibustion, cupping, Chinese therapeutic massage, Qigong, and Tai Chi (exercise therapies that combines postures, movement, and co-ordinated breathing) as well as dietary and lifestyle advice to provide individualized whole-person care after a differential diagnosis. TCM is an energy based system with a holistic approach to the patient encompassing mind, body, and spirit. Today acupuncture is the most widely practiced modality of TCM in the western world.

With increased survivorship rates, many women can have ongoing debilitating side-effects for more than 10 years post-treatment [17,18]. These side-effects may have physical as well as psychological effects on their everyday wellbeing and can include fatigue, hot flushes, anxiety, insomnia, nausea, pain, lymphodema, depression, and weight change [19]. Many women are prescribed chemotherapeutic, estrogen antagonists, or aromatase inhibitors for 5–10 years to prevent recurrence of breast cancer (*tamoxifen, evista, femara, raloxifence, amimidex*) [20]. Currently there seems to be few satisfactory conventional post-treatment strategies for on-going care and support of the specific needs of this group of women. 

Many are turning to TCM therapies, during and as a post-treatment strategy, to alleviate symptoms and to improve their Quality-of-Life (QOL) [4,21,22,23]. Whilst there are many randomized controlled trials on the use of acupuncture for the side–effects of breast cancer treatments, there is little qualitative research to identify and describe motivations for use, attitudes, and perceived benefits of TCM for long-term physical and emotional needs [4,21,22,23]. In Australia, TCM is mostly accessed from the private health sector and occasionally from a cancer support centre at a hospital, but these services are not usually integrated with a hospital [23].

## 2. Materials and Methods 

Qualitative research methods were chosen to open discussions with participants about their choices for breast cancer treatment and post–treatment care. A survey, for example, would not have provided the rich data generated by extended interviews. The value of qualitative data is to support those surviving breast cancer and those who provide care for them not only in considering the likelihood of continued survivorship provided in quantitative studies but also ways to enhance their quality of life. The limited disclosure to oncologists of their patients’ use of CAM therapies and their effects does not advance knowledge of best care in the field.

Thirteen women were recruited via an email sent through Breast Cancer Network Australia in 2014. Initial criteria included being greater than 18 years of age and able to read and write English and having used a TCM modality as part of their treatment journey. Ethics approval was obtained upon application to the Western Sydney University Ethics Review Committee (HREC) H10441. 

### The Participants

The respondents, all living in New South Wales, Australia, had an average age at the time of the interview of 46 years (range from 38 to 64). Eight had dependents living at home. Years since diagnosis ranged from 1 to 11 years (average 4.8 years). All had completed the acute stage of their bio-medical treatment. Many were still suffering side-effects of breast cancer and/or the treatment protocols. All the women had undergone surgery (seven underwent mastectomies), nine received chemotherapy, and ten underwent radiation treatment (Table 1) Eleven of the women took or are taking adjuvant hormone therapies. All experienced symptoms including, but not limited to lymphoedema, fatigue, depression, depressed blood cell count, frozen shoulder, stress, anxiety, hot flushes, and night sweats as a result of their medical treatment. One participant reported recurrence of breast cancer. Six women identified as never having used TCM before (46%). All participants used acupuncture, 62% used Chinese herbal medicine, 23% used Qigong, and 23% used Chinese dietary therapy. (Table 2). None of the women abandoned standard biomedical treatment, but used TCM as an adjunct to standard care. All participants held private health insurance.

Individual in-depth interviews were conducted by one researcher, digitally recorded and transcribed verbatim for analysis. Participants were encouraged to recount their own personal stories and thoughts about their experience with the illness and their use of TCM. All interviews started with a broad open-ended question and continued in a conversational form allowing the women the freedom to talk about their experience. This narrative style focused on what was important to them and allowed entry into their world through their stories. 

Illness narratives identify themes as personal stories which are explored though the women’s treatment and treatment effects [24]. The discussion unfolded why they decided to use TCM, what they valued about the treatment, and any barriers they experienced in using TCM. A question guide (Appendix A) was referred to, covering key concepts identified through the prior literature review. These questions were a guide only and evolved throughout the study. Not all questions were answered by participants. 

A grounded theory formal approach to data synthesis was used, allowing the theory to emerge from the data and to generate new theory [25]. By constant comparison and reflection on the responses to the questions, emergent trends were categorized to identify patterns [25,26]. Each transcript was analysed to identify themes and these were coded [24]. As new themes emerged they were contrasted with previous transcripts to identify key themes [27]. This method was used until saturation of themes was reached.

## 3. Results

The key themes identified included control, relieving, connecting, and constraints in using TCM.

### 3.1. Control 

*Process of Discovery of TCM*—Participants were either current users of TCM who just continued down this path or had multiple information sources as an introduction to TCM. Similar to a previous study on the use of CAM, women were mainly referred by a friend or another breast cancer survivor to a TCM practitioner. Three women received information about TCM use from their oncology team or General Practitioner. This process of self-help and decision-making on the use of TCM engendered a more positive outlook either about the cancer or the symptoms.

“L”—“*Just recommendation from friends who knew other people who had had cancer*”.

“C”—“*My husband had a friend whose wife had been diagnosed twelve months previously. She recommended an acupuncturist/Chinese herbalist*”.

“B”—“*A friend of mine recommended this Doctor of “TCM, so I looked him up and there was a lot of information on the internet about him and cancer treatment*”.

“K”—“*On the advice of my oncologist I started acupuncture and have been going regularly, weekly when first diagnosed. When I was having radiation they said people having acupuncture have a much easier time and I tolerated it well*”.

Physical Disruption and Psychological Suffering after Breast Cancer—The women spoke about bodily changes; loss of fertility; loss of one or both breasts; changes in weight, energy changes, emotional changes, life perspective changes as a result of their illness. Physical loss brings changes in identity, both self-identity and social identity, and coming to terms with the change from healthy and whole to transformed by illness [3]. Physical disruption was a major theme identified in the interviews, and with it the mental suffering for each woman. The desire to be proactive in their health journey and to incorporate physical and psychological factors in one treatment was stressed as a major factor in facilitating their healing process.

“F”—“*It was quite painful for me to do a lot of things—I lost my pectoral muscles so my shoulders would dislocate while I slept. I wasn’t able to help my husband at work, because I couldn’t hold anything. Due to my other chronic health issues a reconstruction was out of the question and I gave up using prosthesis due to skin rashes. After having my ovaries removed and being very slim I put on 15 kg so yes it had an impact in that way*”.

Acupuncture was found to help women with mobility and pain reduction, thereby reducing financial anxiety and feelings of loss.

*Control*—One of the key themes to emerge was empowerment of patients to be pro-active in their care and well-being. TCM treatments were a process of discovery and were found to give the women a locus of control, empowering them to take practical strategies to manage and minimize symptoms. This psychological transformation put them on a positive self-help path and allowed them to “flourish” [28,29,30]. The experiences of transformation brought about positive life changes from work to relationships. This mode of self-help and being an active part of the treatment put them on a positive path, nurtured resilience, and enabled coping [31].

“H”—“*Maybe it is psychological, but I think you are doing something to help yourself by having TCM treatment. The acupuncture treatments were to prevent the cancer coming back and to keep me healthier*”.

*Transformation*—A diagnosis of cancer is a life-changing experience and is often transformational, engendering spiritual and personal growth [32,33,34]. Many of the narratives revealed changes to perceptions and outlooks on life, including work/life balance, friends, and prioritizing themselves before career deadlines. Breast cancer can have positive and negative aspects on life values as women search to find meaning in the disease [35,36]. The women adjusted to new priorities, changed values and life perspectives, and they spoke of prioritizing themselves and having a healthier work/life balance. They felt that the acupuncture gave them a holistic view of growth and development by focusing on mind, body, and spirit.

“B”—“*The whole cancer experience was traumatic, it is a huge shock, maybe a wake-up call. I don’t have such a high-powered job now*”.

“N”—“*I am not willing to put up with a lot of the stresses I used to put up with. I make sure I have time to rest and time for me. Before I would put my health last, but I don’t put work ahead of me anymore*”.

*Illness Outcomes—*Cancer survivors search out complementary care such as TCM because of unmet needs in standard care [37,38]. The desire to be proactive in their health journey and to incorporate physical and psychological factors in one treatment was stressed as a major factor in facilitating their healing process. 

### 3.2. Relieving 

*Relieving to have* “*Time Out*”*—*Some viewed their treatment as a reward, having time to themselves to relax, with a hands-on approach. Taking care-of-self assumed an important role in their lives. Many reported coping better than others during chemotherapy and with health anxiety.

“N”—“*I really enjoy my treatment time. I find it relaxing and meditative to have some ‘me’ time away from the normal stresses*”.

*Relieving Stress*—Participants reported perceived health benefits including anxiety and stress reduction to feeling more balanced and with improved well-being. Psychological distress can occur after the initial diagnosis and continue throughout the treatment process, impacting on tumour progression and affecting the immune system [27,38,39].

“C”—“*I felt it was a great help to get me through chemotherapy and to deal with illness-related stress. My practitioner initially strengthened my immune system and then helped to relieve side-effects of chemotherapy, including nausea*”.

“D”—“*My acupuncture practitioner was a lot more encouraging than the doctors. Honestly, if I didn’t have the acupuncture and Chinese herbs it would be a lot more stressful for me*”.

*Relieving Side-effects of Conventional Treatments*—A range of health benefits were perceived from using TCM, with stress relief and reducing side-effects of standard care cited as the main benefits of use (Table 3). Nausea and low blood count from chemotherapy, fatigue, lymphoedema, hot flushes, insomnia and night sweats, anxiety, and depression had an overwhelming effect on the women, with many symptoms occurring in clusters. Most of the women experienced positive benefits from acupuncture, and no-one reported any adverse effects from their treatment.

“A”—“*It has made a dramatic improvement with side-effects of ‘tamoxifen’ such as nausea, hot flashes, and night sweats as well as my sleep*”.

“F”—“*I had acupuncture to help with hot flashes and night sweats. It definitely helped, and if these symptoms come back I have acupuncture and it completely goes away. I feel more balanced in what I do, I just feel better. I have found it very effective*”.

“G”—“*With my lymphoedema, I had acupuncture if my arm was beginning to ache. The practitioner worked on getting feeling back in the arm. My sternum was hypersensitive too and the oncologist offered me a drug for that! I used acupuncture and Qigong and there has been really quite substantial benefit*”.

“E”—“*I didn’t have a terrible time on chemotherapy. I felt that compared to other’s experience I tolerated it well. My white blood cell count remained strong throughout, my energy levels didn’t drop too dramatically, and I felt my strength return relatively quickly after treatment*”.

“H”—“*I just compared myself to others at the chemo ward. The nurses would say that I was the healthiest-looing chemo patient they ever had. I didn’t have any nausea, my blood count remained good throughout. During the whole treatment I was well, I didn’t lose a lot of weight, I went to the gym twice a week during radiation treatment*”.

“E”—“*When my blood count was getting low my TCM doctor told me what to eat. After the second blood test my red blood cell count had gone up significantly*”.

### 3.3. Connecting 

*Connection in the Therapeutic Relationship*—Many of the women felt more connected to their TCM practitioner with whom they felt they received a more personalized approach in a less hurried atmosphere. The treatment usually involved discussion of emotional aspects of care with attentive listening. Connection with the care provider can be an important factor in the therapeutic relationship.

“D”—“*I found my TCM doctor definitely looks at the emotional side of things and was helpful as someone to talk to. He was more encouraging than the other doctors about moving forward*”.

“E”—“*My TCM doctor made me comfortable and it felt a bit like therapy as well as treatment—counselling therapy, because we would talk. It was a marked difference to the chemotherapy suite at the hospital*”.

*Mind Body Spirit Connection*—Others took up Qigong to establish a mind-body connection and to improve spiritual and physical wellbeing and reduce stress. Qigong involves specific breathing techniques combined with a mindful focus and specific movement techniques to manipulate the body’s qi (energy) to improve physical and spiritual well-being. This re-connection with the body and spirit is important for general well-being and QOL [40,41,42].

“K”—“*I discovered Qigong at a retreat and found it to be absolutely wonderful*”.

“G”—“*I was lucky to find someone who teaches medical Qigong. I do this meridian breathing and it’s really powerful for my energy*”.

Evidence suggests that physiological stress is improved with techniques of meditation, Qigong, and tai chi. In a study conducted by Lee et al. (2006), 35 women with breast cancer and undergoing chemotherapy experienced improved QOL, less fatigue, and improvement in mood and psychological distress after attending Qigong training during their chemotherapy treatments [40].

*Connection between Bio-medical Care and TCM/Polarization and Integration*—Many of the women expressed a desire to have a more connected approach and effective communication regarding other treatments that may help their side-effects. Negativity from some of their bio-medicine doctors regarding TCM caused anxiety, conflict, and barriers for some of the women. They indicated that it would be beneficial for a combined approach instead of feeling “caught between two worlds” [43,44,45]. None of the women were advised to discontinue use of acupuncture, but most were deterred in their use of Chinese herbal medicine, particularly during radiation treatment.

“H”—“*Everything should be provided with the oncologist and radiologist—it makes more sense to integrate—the TCM doctor, massage, the naturopath—it should be together and done together, because you are trying to make someone better. Germany is the classic case, they all work together there*”.

“E”—“*I feel like I suffered a little bit, by being caught between two worlds. The conflict of points of view, the whole kind of evidence debate, the scenario where there are not enough studies to support and to argue in an absolutely convincing way with the oncologist what I wanted to do—where it can be proven and backed up with scientific evidence. I feel that the acupuncture did support me and it was the bare minimum of what the oncologist would allow and I feel that it takes a bit of strength to pursue beyond that*”.

“G”—“*He (the oncologist) just rolled his eyes when I told him about the herbs. They were ok about it. He said it would be different if I was having chemotherapy because you don’t want anything that interferes with that or would be counterproductive to what they are doing*”

### 3.4. Constraints in Using TCM

Faced with disruption to their lives, the women used the resources available to them to manage their illness. Most found it was easy to have TCM treatment, as it was available close to where they lived and they were covered by their private health insurance, so short-term use was not a problem. Financial and time constraints were cited as the two biggest hindrances in using TCM long-term or after a return to work.

“B”—“*I continued treatment with acupuncture and Chinese herbal medicine for 18 months. The cost was huge, it was on the other side of town so the time and financial constraint had a bearing on stopping*”.

“I”—“*I am able to claim on my private health fund. With cancer treatment everything is expensive, you just find the money*”.

“L”—“*I haven’t been for three weeks. I’ve run out of time, as I have gone back to work and have two children. It’s probably more the logistics of getting there after work*”.

“K”—“*Absolutely no limitations. Benefits are paid from my private health fund, no problem getting to appointments*”.

## 4. Discussion

Thirteen women were interviewed. All the women had undergone surgery (seven underwent mastectomies), nine received chemotherapy, and ten underwent radiation treatment. Eleven of the women took or were still taking adjuvant hormone therapies. One participant reported recurrence of breast cancer. In this study 54% of the women were already users of TCM, 23% were referred by friends, and 23% were referred by their doctors. 

All participants reported being satisfied with the treatment received from their TCM practitioners. The patient-TCM practitioner therapeutic relationship was an important part of the treatment process. Many of the women developed an ongoing relationship with their practitioner probably due to, (a) longer treatment time; (b) comfortable and relaxing atmosphere; (c) more personal care; (d) able to talk about their worries in an open way. The concept that the women had a greater benefit from having the TCM treatment in a different setting to the hospital setting is one that needs further research. Integrating TCM into the bio-medical system may reduce the benefit when the setting is not as therapeutic or results in “white coat syndrome” [43].

It is evident from this small pilot study that there are commonalities between users of TCM. They used TCM for physical and emotional issues in the treatment and post-treatment stages with the most common reasons being to reduce stress, fatigue, pain, hot flushes, and insomnia, increase general well-being and QOL, to enable coping, to prolong life expectancy, and boost immunity (Table 3). With 69% of the participants using TCM to reduce stress, the treatments were valued in addressing their psychological needs as well as for reduction of treatment related fatigue (54%). Having the treatments gave them more control of their bodies and the illness, giving them a more positive outlook. Some of the participants reported cost, logistics of getting to appointments, and time as barriers that prevented them from continuing with the TCM long-term.

Similar to other studies, the interviewees expressed a desire for more information from physicians [42]. Effective communication between oncologists and patients is needed to reach mutually informed decisions, to respect patients’ preferences, and to integrate safe TCM practice as part of their care. Oncologists and their associated team need to be aware of the physical and emotional needs of their patients and to be able to provide informed advice on TCM modalities that may be of benefit for patient well-being [42,43,44,45]. 

The women benefited from their TCM treatments and reported improved QOL during treatment and in their transition from treatment stage to survivorship. They found their use of TCM gave them a sense of responsibility for their health and well-being, and rather than put up with treatment side-effects they sought to alleviate these problems by taking practical strategies to manage and minimize symptoms. This mode of self-help coupled with being an active part of the treatment put them on a positive path, nurtured resilience, and enabled coping [33,34]. 

## 5. Conclusions 

TCM is becoming increasingly popular for breast cancer patients [45]. Effective communication between biomedical doctors and patients is essential to integrate safe TCM practice as part of their care. The women benefited from their TCM treatments, reporting improved QOL during treatment stages and in their transition from treatment stage to survivorship.

This study found that the motivations, objectives, and benefits of TCM use in breast cancer survivors exhibited commonalities. They used TCM in a supportive role for physical and emotional issues in the treatment and post-treatment stages. With 69% using TCM to reduce stress, the treatments were valued for addressing their psychological needs and reducing treatment related fatigue (54%). The women continued to use TCM regardless of whether their oncology team approved their use or not. They were either referred by a friend or read about TCM and they made their decision from there. They reported a need for their oncology team to be more willing to work with their TCM practitioner.

This study had some limitations. The sample size was predominantly from the Sydney area and of Caucasian background. All transcriptions were coded by one researcher and checked by another researcher. Conducting a larger study with more geographical and cultural diversity would improve generalizability of results. This further research will determine if the emerging themes are representative of women’s experiences of using TCM as part of their breast cancer journey. Integrative oncology is gaining momentum in western countries with a focus on individualized, patient-oriented care that addresses more than symptoms. The unmet needs of these participants and other cancer survivors could be a future meeting point between the two systems. 

This article provides an introduction, as described by breast cancer survivors, on their reasons and benefits of using TCM as part of their treatment journey.

## Figures and Tables

**Table 1:** Profile of participants.

Research Participant	Age at Diagnosis	Current Age	Marital Status	Type of Cancer	Treatment	Special Characteristics or Circumstances
A	52	58	De-facto	DCIS	Mastectomy, lymph node removal, chemo, tamoxifen, aromatase inhibitors	
B	44	52	Single with dependants	DCIS/EBC	Mastectomy, lymph node removal, chemo, radio, tamoxifen	Frozen shoulder from radiation treatment, breast reconstruction which had to be re-done
C	56	58	Married with dependants	Lobular Carcinoma in Situ	Mastectomy, chemo, radio, lymph node removal, femara	Other breast removed year later and reconstruction
D	49	53	Single	EBC	Lumpectomy, lymph nodes removed, radio, aromatase inhibitors	
E	44	47	Married with dependants	DCIS/EBC	Mastectomy, chemo, radio, tamoxifen	
F	44	55	Married with dependants	EBC, Grade 4, invasive	Bilateral mastectomy, lymph node removal, ovaries removed	Frozen shoulder, health complicated by long-term lupus & thyroidectomy
G	50	51	Married with dependants	Infiltrating DC x 2, Grade 2	Mastectomy, lymph node removal, radiation, tamoxifen, femara	Secondary cancer in sternum and spine
J	58	62	Married	EBC	Mastectomy, lymph node removal, chemo, tamoxifen, aromatase inhibitors, femara	
K	64	67		EBC	Wide local excision, lymph node removal, chemo, radio, tamoxifen, aromatase inhibitors	
L	38	39	Married with dependants	EBC Stage 3	Surgery, chemo, radio, tamoxifen	22 weeks pregnant when diagnosed, ovaries removed at a later date
M	60	70	Single	DCIS	Lumpectomy, lymph node removal, radio, tamoxifen	
N	43	46	Married	EBC	Lumpectomy, lymph node removal, chemo, radio, tamoxifen, aromatase inhibitors	Frozen shoulder caused by radiation treatment

Chemotherapy—chemo; Radiation Treatment—radio; Tamoxifen, Femara, Aromatase inhibitors—Adjuvant pharmaceutical treatment used for 5–10 years post-diagnosis; DC—Ductal carcinoma; DCIS—Ductal carcinoma in situ; EBC—Early breast cancer.

**Table 2:** Modality of Traditional Chinese Medicine (TCM) used.

Modality Used	Number of Patients	Percentage of Patients
Acupuncture	13	100%
Chinese Herbal Medicine	8	62%
Qigong	3	23%
Diet Therapy	3	23%

**Table 3:** Patient concerns.

Patients Concerns	Number of Patients	Percentage of Patients
Stress and coping	9	69%
Fatigue	7	54%
General well-being	5	38%
Adjuvant therapy side-effects	5	38%
Chemotherapy side-effects	4	31%
Radiation side-effects	4	31%
Hot Flushes	4	31%
Pain	3	23%
Boost immunity	2	15%
Insomnia	2	15%
Night sweats	2	15%
Prevent recurrence	1	8%

## Appendix A

Questionnaire Content (These questions are a guide only)

1. What impact did your diagnosis have on your Quality of Life?
  *Probe—*Physical, spiritual/emotional, financial, work/home life
2. Had you used TCM before you were diagnosed?
3. If you hadn’t used it before, how did you find out about TCM?
4. How did your experience with breast cancer lead to use of Traditional Chinese Medicine (TCM)?
  *Probe—*At what stage did you use TCM?
5. Did you use any other complementary therapies?
6. What type of TCM therapies did you seek/receive?
7. What do you experience as the benefits/limitations of TCM therapies?
  *Probe—*Tell me a little more about it?
8. Did you experience any barriers to using TCM?
  *Probe*—Time, distance, financial?
9. What reaction did you receive from your doctors that you were using TCM?
10. How did you experience interactions with the different practitioners you have been involved with and how important have these been for your experience of having cancer?
  *Probe—*What did you think or feel about?
11. What advice would you give someone, newly diagnosed with breast cancer, based on your experience?
12. Do you have any other thoughts about breast cancer you would like to share?
